# Liberia Medical School

**Published:** 1845-03

**Authors:** 


*Liberia Medical School.*—In a letter from Dr. J. W. Lugenbeel, the colonial physician in that interesting and rising state, Monrovia, Africa, to the Secretary of the American Colonization Society, under date of Oct. 22d, he acknowledges the receipt of sixteen volumes of medical books, the gift of Dr. Bell, of Philadelphia, "for the use of the Liberia Medical School." He further observes, "My students are making fine progress in their studies. They are of very considerable assistance to me, and I hope and believe they will become blessings to the colony. I endeavor to give them every opportunity to learn practically, as well as theoretically, by frequently taking them with me and giving them clinical lectures." From some further remarks in the same communication, we learn that Dr. Lugenbeel is decidedly of the opinion, that ninety-nine persons in a hundred, visiting Africa from America, might pass safely through the acclimating fever of the country, provided their constitutions were not much impaired by previous disease, and they could be prevailed on to exercise that prudence which is necessary. Moderation in exposure and exercise, contentment of mind, and temperance in eating and drinking, and in the *use of physic*," he says, "are *sine qua nons* in this country." Without doubt, the discovery will hereafter be announced that there is necessarily no acclimating fever there. This notion is already beginning to attract attention. The bad state of preparation by the mode of living on the voyage, unquestionably predisposes to the developement of bilious and congestive fevers, on landing in the new settlements. We consider that Dr. Lugenbeel's observations are already tending to this opinion, which is a favorite theory, at least with ourselves.—*Boston Med. and Surg. Jour.*

